# Mechanical Ventilation Exacerbates Poly (I:C) Induced Acute Lung Injury: Central Role for Caspase-11 and Gut-Lung Axis

**DOI:** 10.3389/fimmu.2021.693874

**Published:** 2021-07-19

**Authors:** Shuqing Jin, Xibing Ding, Chenxuan Yang, Wenbo Li, Meihong Deng, Hong Liao, Xin Lv, Bruce R. Pitt, Timothy R. Billiar, Li-Ming Zhang, Quan Li

**Affiliations:** ^1^ Department of Anesthesiology, Shanghai Pulmonary Hospital, TongJi University, Shanghai, China; ^2^ Department of Surgery, University of Pittsburgh Medical School, Pennsylvania, PA, United States; ^3^ Department of Anesthesiology, Renji Hospital, Shanghai Jiaotong University Medical School, Shanghai, China; ^4^ Department of Breast Surgical Oncology, National Cancer Center/National Clinical Research Center for Cancer/Cancer Hospital, Chinese Academy of Medical Sciences and Peking Union Medical College, Beijing, China; ^5^ Department of Surgery, The Ohio State University, Ohio, OH, United States; ^6^ Department of Environmental Occupational Health, University of Pittsburgh Graduate School Public Health, Pennsylvania, PA, United States; ^7^ Department of Anesthesiology and Perioperative Medicine, University of Pittsburgh School of Medicine, Pennsylvania, PA, United States; ^8^ Department of Anesthesiology, National Cancer Center/National Clinical Research Center for Cancer/Cancer Hospital & Shenzhen Hospital, Chinese Academy of Medical Sciences and Peking Union Medical College, Shenzhen, China

**Keywords:** acute lung injury, Poly(I:C), mechanical ventilation, gut-lung axis, systemic endotoxemia, caspase-11, caspase-1, pyroptosis

## Abstract

**Background:**

The mechanisms by which moderate tidal volume ventilation (MTV) exacerbates preexisting lung injury are unclear. We hypothesized that systemic endotoxemia *via* the gut-lung axis would lead to non-canonical and canonical inflammasome activation and pyroptosis in a two-hit model involving polyinosinic-polycytidylic acid (Poly(I:C)), a synthetic analog of dsRNA and MTV and that this would associate with acute lung injury (ALI).

**Methods:**

Anesthetized mice were administered Poly(I:C) intratracheally and then 6 h later, they were mechanically ventilated for 4 h with otherwise non-injurious MTV (10ml/kg). Changes in intestinal and alveolar capillary permeability were measured. Further documentation of ALI was assessed by evans blue albumin permeability, protein and IL-1 family concentration in bronchoalveolar lavage fluid (BALF) or plasma, and histopathology in cohorts of wildtype (WT), whole body genetically ablated caspase-11 (caspase-11^-/-^), caspase-1/caspase-11 double knockout (caspase-1/11^-/-^), gasdermin D (GSDMD)^-/-^, nucleotide-binding domain leucine-rich repeat-containing protein 3 (NLRP3)^-/-^ and advanced glycosylation end product-specific receptor (RAGE) ^-/-^ mice.

**Results:**

Non-injurious MTV exacerbated the mild lung injury associated with Poly(I:C) administration. This included the disruption of alveolar-capillary barrier and increased levels of interleukin (IL)-6, high mobility group proteins 1 (HMGB-1), IL-1β in BALF and IL-18 in plasma. Combined (Poly(I:C)-MTV) injury was associated with increase in gastrointestinal permeability and endotoxin in plasma and BALF. Poly(I:C)-MTV injury was sensitive to caspase-11 deletion with no further contribution of caspase-1 except for maturation and release of IL-18 (that itself was sensitive to deletion of NLRP3). Combined injury led to large increases in caspase-1 and caspase-11. Genetic ablation of GSDMD attenuated alveolar-capillary disruption and release of cytokines in combined injury model.

**Conclusions:**

The previously noted exacerbation of mild Poly(I:C)-induced ALI by otherwise non-injurious MTV is associated with an increase in gut permeability resulting in systemic endotoxemia. The gut-lung axis resulted in activation of pulmonary non-canonical (cytosolic mediated caspase-11 activation) and canonical (caspase-1) inflammasome (NLRP3) mediated ALI in this two-hit model resulting in GSDMD sensitive alveolar capillary barrier disruption, pyroptosis (alveolar macrophages) and cytokine maturation and release (IL-1β; IL-18). Pharmacologic strategies aimed at disrupting communication between gut and lung, inhibition of inflammasomes or GSDMD in pyroptosis may be useful in ALI.

## Introduction

Sepsis is the major underlying cause (~75%) of acute respiratory distress syndrome (ARDS) and this often follows the onset of pneumonia (1). ARDS also occurs in a large number of patients from infections outside the lung and the mechanisms underlying the development of lung injury from remote sources are multifactorial and poorly understood (2). Both direct (e.g. pneumonia) and indirect (extrapulmonary) sepsis routinely require mechanical ventilation and it is well known that such lifesaving therapy can exacerbate underlying lung injury in an iatrogenic pathology of ventilator induced lung injury [VILI; (3)]. Indeed, minimizing over distension (volutrauma) and/or alveolar collapse and reopening (atelectrauma) by lung-protective ventilation (4) has had an impact in reducing morbidity and mortality from ARDS.

In preclinical studies, sensitization of VILI to preexisting acute lung injury (ALI) secondary to pneumonia (5, 6), intratracheal endotoxin (7–10), viral (11) and sterile injury (12–14) is apparent. Although preclinical outcomes vary as a function of magnitude of extrapulmonary septic condition and the nature of mechanical ventilation parameters [tidal volume, onset and duration; positive end expiratory pressure; (15)], sensitization of VILI to events originating in distal site and plasma space including exogenous endotoxin (16, 17) and polymicrobial sepsis (18, 19) has also been documented.

In the current study, we approached connections of direct and indirect lung injury that are predicted from possible gut-lung interactions and sensitization to subsequent non-injurious moderate mechanical tidal volume ventilation (MTV). We (20) and others (21) have shown that MTV can exacerbate lung injury after intratracheal delivery of polyinosinic-polycytidylic acid [Poly(I:C)], a synthetic analog of dsRNA (that itself can be produced by many viruses during their replicative cycle). The gut plays an important role in indirect lung injury by releasing infectious microbes and inflammatory, injurious mediators directly into the circulation or *via* the lymph system secondary to enhanced gut permeability (22). Endotoxin [lipopolysaccharide (LPS)] derived from gram negative microbes in the gut may thus be liberated in large amounts in the circulation and contribute to lung injury (23, 24). The canonical detection mechanism of LPS occurs *via* cell-surface toll like receptor-4 (TLR4), however but it is noteworthy that lung injury after Poly(I:C)-MTV (21) or systemic endotoxemia (25) involve TLR4 independent mechanisms. In this latter comprehensive study (25), the authors noted that indirect lung injury due to systemic endotoxemia involved non-canonical inflammasome caspase-11 mediated pyroptosis, an inflammatory programmed cell death, in pulmonary endothelium of intact mice. Murine caspase-11 is the cytosolic receptor for LPS. Activation of caspase-11 by LPS leads to cleavage of gasdermin D (GSDMD) and the N-terminal cleavage fragment (GSDMD-N) leads to cell permeabilization and pyroptosis (26).

Accordingly, we: a) confirmed that pretreatment of intact mice with intratracheal (i.t.) Poly(I:C) would lead to sensitization to ALI due to otherwise non-injurious MTV; and b) hypothesized that ALI after Poly(I:C)-MTV was associated with gut derived LPS and caspase-11 non-canonical inflammasome mediated pyroptosis.

## Materials and Methods

### Experimental Protocols

The Animal protocols were approved by the Animal Care and Use Committee and experiments were performed in strict adherence to NIH Guidelines and followed current guidelines for preclinical models in research. Protocols (with brief descriptions below) included: a) MTV enhanced Poly(I:C) induced ALI is associated with increased gastrointestinal permeability and increased endotoxin in plasma and lung; b) MTV exaggerates Poly(I:C) induced acute lung injury through a caspase-11 dependent process; and c) Regulation of caspase-11 expression and activation, its relationship to canonical nucleotide-binding domain leucine-rich repeat-containing protein 3 (NLRP3) mediated caspase-1 activity and gasdermin cleavage dependent pathways in whole lung and isolated macrophages after Poly(I:C), MTV and their combination in intact mice.

### *In-Vivo* Experimental Animal Model

C57 BL/6 mice (8-10 weeks old, male) were purchased from Jackson Laboratories. Caspase-1/11-/- mice, caspase-11-/- mice, TLR4-/- mice, NLRP3-/- mice, GSDMD-/- mice, advanced glycosylation end product-specific receptor (RAGE)-/- mice were bred and maintained in the University of Pittsburgh animal facility according to NIH animal care guidelines and all procedures were performed according to University of Pittsburgh Animal Research Protocols. A total number of 168 wild-type (WT) mice, 88 Caspase-1/11-/- mice, 88 Caspase-11-/- mice, 32 NLRP3-/- mice, 40 GSDMD-/- mice, 16 RAGE-/- mice were used in this project.

The animal model protocols was as follows: Mice were prospectively randomized to one of four groups (n=4-12 per group): (a) SHAM: 100 µL endotoxin free water intra-tracheal 10h before the experimental endpoint with spontaneous breathing; (b) Poly(I:C): 3 mg/kg intra-tracheal Poly(I:C) (tlrl-picw, InvivoGen, USA), with spontaneous breathing for 10h; (c) MTV: 6 h after receiving a volume of 100µL intra-tracheal endotoxin free water then connected to a rodent ventilator, and ventilated for 4 h with tidal volume of 10 mL/kg, positive end-expiratory pressure of 0 cm H_2_O, FiO_2_ 0.21, 140 breaths/min; and (d) combined Poly(I:C)-MTV: 6h after an intra-tracheal dose of 3 mg/kg Poly(I:C), mice were ventilated for 4h before harvest. The ventilator parameters were the same of that in MTV group. In all groups, ketamine and xylazine were used to maintain anesthesia. Mean arterial blood pressure, heart rate and oxygen saturation were recorded using a mouse STARR system (Life Science Co.). Mice were sacrificed (10h after starting each protocol) by injecting peritoneal pentobarbitone 300 mg/kg. Additional details were previously described (20).

Additional cohorts of wildtype, Caspase-1/11-/- and Caspase-11-/- mice were prospectively randomized to same four groups as above. Lung tissue and freshly cultured primary alveolar macrophages were obtained for determination of pro- and cleaved caspases-11 and -1.

In addition, wildtype mice were prospectively randomized to these same four groups. Water bottles were removed from cages in the morning and 100 mg/mL FITC-D (4 kD) in PBS was administered (44 mg/100 g body weight) by oral gavage 4 hours before sacrifice. After 4 hours, anesthetize the mice by injecting peritoneal pentobarbitone and collect the blood using 1 ml syringe with 25 G needle by cardiac puncture. Blood or bronchoalveolar lavage fluid (BALF) was placed in microtainer tubes in the dark. Once blood and BALF have been collected from all the mice and all the samples was placed at 37°C for more than 1 hour, tubes were processed to centrifuge for 10 minutes at 800 grams. Concentration of fluoresceine isothiocyanate (FITC) in serum of BALF determined spectrophotofluorometrically (excitation 485 nm; emission 528 nm). A standard serially diluted FITC-Dextran (0 to 8 µg/mL) was used. Serum from mice not injected with FITC-D was used as blank. In addition, endotoxin was measured (LAL Chromogenic endpoints assay, Hycult biotech, PA, USA) in serum and BALF of wildtype mice in these four groups.

An additional cohort of wildtype were assigned to four protocols above and at time of sacrifice, alveolar macrophages (AMs) and neutrophils, lymphocytes were obtained *via* bronchoalveolar lavage for short term culture and immunofluorescence staining. In brief, mice were bled by cardiac puncture and a catheter (20 G) was inserted into tracheal and connected to 1 mL syringe filled with phosphate buffered saline [PBS (Ca^2+^/Mg^2+^ free)]. A total of 5 mL of PBS was used to wash lungs (10x) and the lavage fluid was centrifuged (600 g, 10 minutes) at 4°C. The diluted cells were distributed on cell-counting plates and counted under a microscope. For differential cell sorting, cells were stained with Wright-Giemsa reagents (Baso, Zhuhai, China). The number of neutrophils, macrophages, and lymphocytes per 200 cells was determined based om morphology. Otherwise the diluted cells were resuspended in Roswell Park Memorial Institute [RPMI (2x10^6^ cells/mL)] with 12% serum and transferred to 35 mm petri dishes with 10 mm microwells (Mat Tek corp, Ashland, MA) and placed in incubator for 2 h to extract AMs. Media was changed and adherent cells (e.g. enriched in alveolar macrophages) were assessed for caspase-1 (FAM-FLICA^®^ Caspase-1 Assay Kit (ImmunoChemistry Technologies, ImmunoChemistry Technologies, LLC), pyroptosis (*In Situ* Cell Death Detection Kit, TMR red (Sigma-Aldrich), and nuclear staining (Bisbenzimide Hoechst 33258) Imaging was observed and recorded with 600× magnification using a Olympus confocal microscope.

### Alveolar-Capillary Permeability

Evans blue (Sigma-Aldrich) albumin (EBA; 0.5%, 25 mg/kg) was injected intravenously 1 h before euthanasia and lung harvesting. Blood samples and lung tissue were obtained and processed as described previously (18–20) and EBA permeability was calculated by dividing pulmonary EBA absorbance at 620 nm/g lung tissue by plasma EBA absorbance at 620 nm.

### Histological Examination

For Hematoxylin & Eosin (H & E) staining, the left upper lobe was inflated with 4% paraformaldehyde, embedded in paraffin and assessed *via* semiquantitative histopathology at light microscopic level including following features: edema, hyperemia and congestion, neutrophil margination and tissue infiltration, intra-alveolar hemorrhage and debris, and cellular hyperplasia (18). Each feature was graded as absent, mild, moderate, or severe, with a score of 0-3.

### Western Blot Analysis

Cell lysis buffer (cell signaling technology) and a cocktail of protease inhibitors (Sigma-Aldrich) were used to extract protein in lung tissues and alveolar macrophages. 12% SDS gels was used for electrophoresis, Electrophoresis was performed at 80 V for 120 minutes. Then the protein in gels was transferred for 120 minutes at 200 mA to nitrocellulose membranes. 5% milk in 1% Tween-20 in PBS was used to block membranes. The membranes were incubated with a primary antibody (anti-caspase-11 polyclonal antibody, 1:1000, abcam; anti-caspase-1 polyclonal antibody, 1:1000; abcam) at 4°C overnight and washed three times with PBST (0.1% Tween-20 in PBS). Secondary antibody (1:5000; InvivoGen, USA) was then added and incubated at 37°C for 1 h.

### Cytokines

IL-1β (ELISA, R&D), IL-6 (ELISA, R&D), damage associated molecular pattern molecules (HMGB1, ELISA, Shino-test Corporation) concentrations in BALF and IL-18 in plasma (ELISA, Medical and Biological Laboratories CO., LTD) were determined by enzyme-linked immunosorbent assay (ELISA) according to the manufacturer’s instructions.

### Statistical Analysis

Statistical analysis was performed in GraphPad PRISM 7 (Graph Pad Software Inc.). All data were presented as the means ± Standard Error of Mean (SEM). Data were analyzed by one-way analysis of variance (ANOVA) and Student-Newman-Keuls test if normally distributed. Mann-Whitney U-test was used for analyzing nonparametric data. In figures asterisks denote statistical significance (*p < 0.05; **p < 0.01; ***p < 0.001).

## Results

### MTV Enhanced Poly(I:C) Induced ALI Is Associated With Increased Gastrointestinal Permeability and Increased Endotoxin in Plasma and Lung

**Figure 1:**
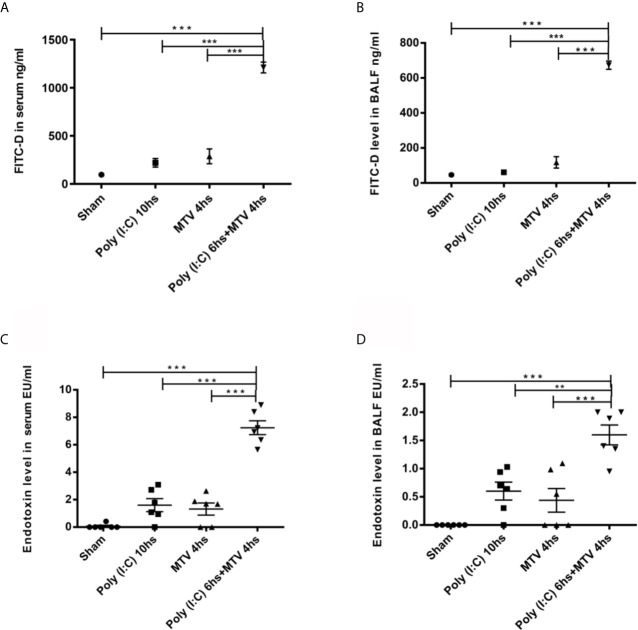
Intestinal and alveolar-capillary permeability. Intestinal permeability of mice evaluated by FITC-D levels in serum **(A)** and in bronchoalveolar lavage fluid (BALF) **(B)** 200 µL FITC-D (30 mg/mL) was instilled through orogastric feeding. Endotoxin levels in serum **(C)** and in BALF **(D)** were measured. All PBS and Poly(I:C) used were endotoxin-free. Mice were divided into four groups treated with Sham as control, Poly(I:C), mechanical ventilation with tidal volume of 10mL/kg (MTV) and Poly(I:C) followed with MTV as indicated in the figure. Results are shown as means ± SEM (n=6) and compared by one-way ANOVA and Student-Newman-Keuls test. **p < 0.01, ***p < 0.001.

**Figure 2:**
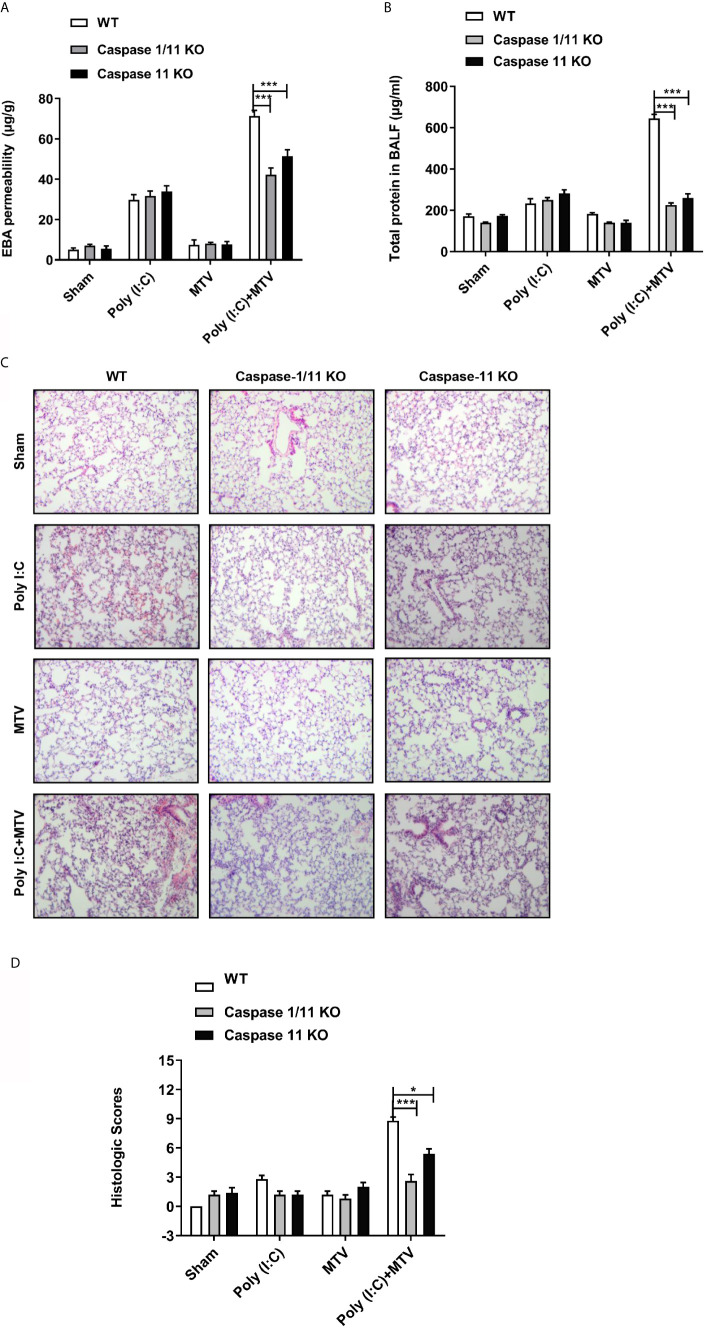
Caspase-1 and caspase-11 prevented Poly(I:C)-MTV induced lung injury. Wildtype (WT), Caspase-1/11 null and Caspase-11 null mice (Caspase 1/11 KO and Caspase 11 KO) were treated with four groups as indicated. EBA permeability (n=5) **(A)**, total protein concentration in BALF (n=6 for WT mice, n=12 for caspase-1/11 and caspase-11 KO mice) **(B)**, H&E histology (n=5) **(C)** of the lung cross section from WT, caspase-1/11 KO, caspase-11 KO mice (Scale bars: 50 μm) and total histopathologic scores of lung injury **(D)** were evaluated by two different authors calculated for each animal. Results are shown as means ± SEM and compared by one-way ANOVA and Student-Newman-Keuls test. *p < 0.05, ***p < 0.001.

Patients with severe respiratory viral infection may require ventilation and Poly(I:C) is a double stranded RNA immune stimulant used to mimic the immune activation of viral infections (27, 28). We (20) and others (21) have previously shown that Poly(I:C) pre-treatment markedly increases lung injury induced by moderate tidal volume ventilation (MTV=ventilation at 8-10 mL/kg). We assessed intestinal and alveolar-capillary permeability in Poly(I:C)-MTV mice by measuring the transmigration of FITC-D (4 kD) from gastrointestinal contents into plasma (**Figure 1A**) and BALF (**Figure 1B**) spaces, respectively. There were modest increases in both organs after either stimulus alone but the combination of 3 mg/kg intra-tracheal Poly(I:C) for 6 h followed by MTV for 4 h led to large increases in both gastrointestinal permeability and alveolar-capillary permeability to FITC-D. We then measured endotoxin in plasma (**Figure 1C**) and BALF (**Figure 1D**) and noted small increases after either stimulus alone but large increases in endotoxin in plasma and BALF after combined Poly(I:C)-MTV. It is unlikely that endotoxin was due to contamination of Poly(I:C) as the solution tested negative prior to intratracheal instillation. Further refinement and quantitation of lung injury (EBA permeability, BALF protein, histology, cytokine release) was used to assess the nature of interaction of Poly(I:C) and MTV in **Figure 2**.

Increased levels of circulating and intrapulmonary endotoxin after combined injury prompted us to pursue potential role of the intracellular endotoxin receptor caspase-11 (TLR4-independent) in acute lung injury.

### MTV Exaggerates Poly(I:C) Induced Acute Lung Injury Through a Caspase-11 Dependent Process

Viral infection can activate interferon responses and this can promote the up regulation of the caspase-11 non-canonical inflammasome (27). To determine if up regulation of caspase-11 in the lungs contributed to the pulmonary response to ventilation, we pre-treated wild-type and caspase-11-/- mice with Poly(I:C) followed 6 h later with MTV for 4 h. Caspase-11 activation can promote the activation of the caspase-1 canonical inflammasome (29, 30). Therefore, to determine the relative contribution of caspase-1 to the injury response we also included mice deficient in both caspase-1 and caspase-11. As shown in **Figure 2** [and consistent with the above findings (**Figure 1**) and our previous findings (18–20)], MTV alone for 4 h had no impact on indices of lung injury including leakage of Evans blue dye into the lung (**Figure 2A**), accumulation of protein into the BAL fluid (BALF; **Figure 2B**), or histologic scoring of lung injury (**Figures 2C, D**). While Poly(I:C) treatment alone induced modest increases in Evans blue dye (**Figure 2A**) and protein accumulation (**Figure 2B**) in the BALF, the initiation of MTV at 6 h after Poly(I:C) markedly increased the appearance of these large molecular weight species as well as histopathologic quantitative assessment of nature of ALI (**Figure 2D**). The deletion of caspase-11 had no impact on the mild lung injury induced by Poly(I:C) alone but almost completely prevented the exaggerated injury induced by the addition of MTV to Poly(I:C). No further protection was seen in mice deficient in both caspase-11 and caspase-1 consistent with caspase-11 being central to acute lung injury, induced by sequential hits.

**Figure 3:**
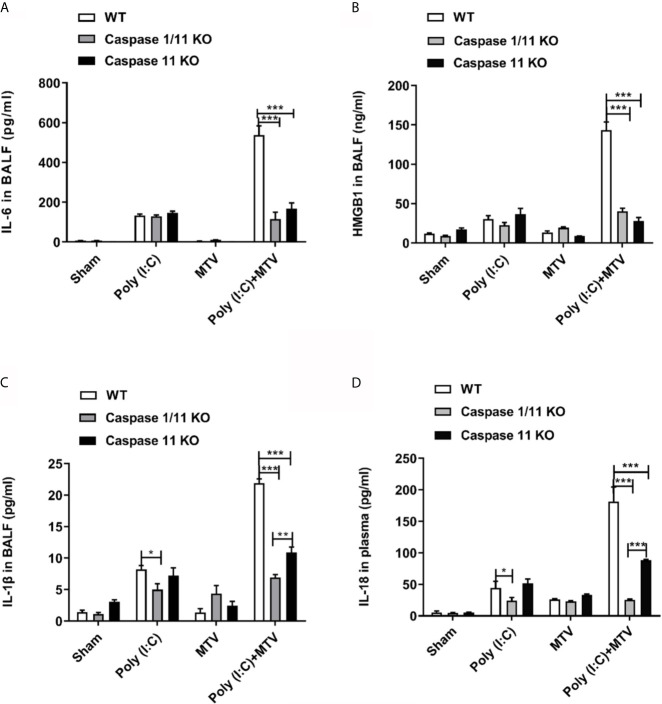
Caspase-1 and caspase-11 alleviated Poly(I:C)-MTV induced pulmonary informatory response. IL-6 **(A)**, HMGB1 (n=5) **(B)** and IL-1β **(C)** in BALF as well as IL-18 levels **(D)** in plasma were significantly decreased in Caspase 1/11 KO and Caspase-11 KO mice compared to WT mice. In **(A, C, D)**, n=6 for WT mice, n=12 for caspase-1/11 and caspase-11 KO mice. Results are shown as means ± SEM and compared by one-way ANOVA and Student-Newman-Keuls test. **p < 0.01, ***p < 0.001.

To assess the requirement for caspase-11 and caspase-1 on inflammatory mediator production, IL-6, HMGB1 and IL-1β were measured in BALF (**Figures 3A–C**) and IL-18 in plasma (**Figure 3D**). Similar to the observations made on lung injury, the addition of MTV to Poly(I:C) significantly increased levels of IL-6, IL-1β and HMGB1 in the BALF and IL-18 in the plasma. Deletion of caspase-11 alone or caspase-11 and caspase-1 together significantly suppressed the increases in these mediators induced by MTV+Poly(I:C). A significant difference in the degree of mediator suppression between the mouse strains was seen for BALF IL-1β levels and plasma IL-18 levels, where deletion of both caspase-11 and caspase-1 lead to an even greater decrease in IL-1β and IL-18 levels than that seen with deletion of caspase-11 alone (**Figure 3D**). These findings indicate that caspase-1 contributes to IL-6, IL-1β and IL-18 release into the circulation.

### Regulation of Caspase-11 Expression and Activation, Its Relationship to Caspase-1 Pathways in Whole Lung and Isolated Macrophages After Poly(I:C), MTV and Their Combination in Intact Mice

**Figure 4:**
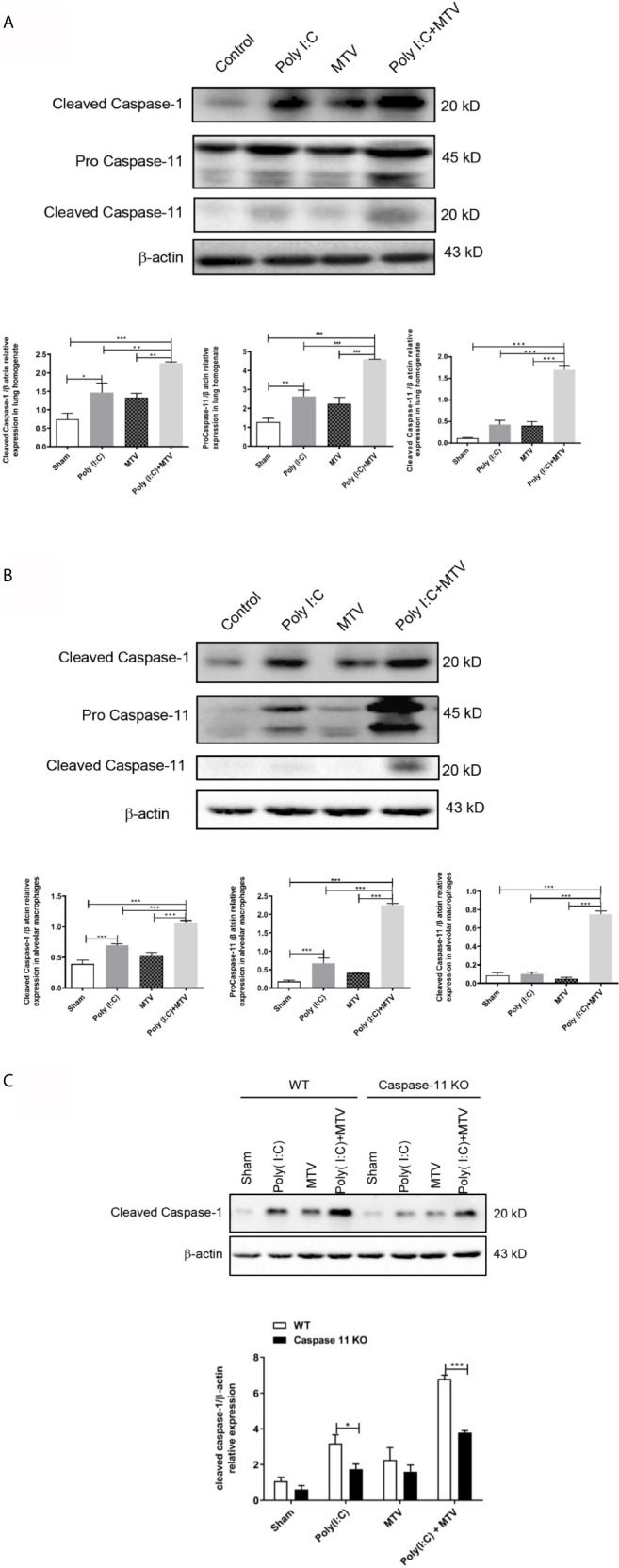
Alterations of protein levels of cleaved caspase-1, procaspase-11 and cleaved caspase-11 following Poly(I:C)-MTV. MTV following Poly(I:C) instillation resulted in a significant increase in procapase-11 and, large increase in appearance of cleaved caspase-11 in whole lung **(A)** and alveolar macrophages **(B)**. Cleaved caspase-1 expression levels were inhibited in caspase-11 KO mice in Poly (I:C)-MTV compared to that in WT mice **(C)**. Results are shown as means ± SEM (n=12) and compared by one-way ANOVA and Student-Newman-Keuls test. *p < 0.05, **p < 0.01, ***p < 0.001.

We quantified expression and activation of caspase-11 and caspase-1 in whole lungs and freshly isolated alveolar macrophages by Western blot after Poly(I:C) and/or MTV. Compared to the control group, there was a slight increase of procaspase-11 after Poly(I:C) administration while procaspase-11 expression was not affected by MTV alone. MTV after Poly(I:C) priming resulted in a significant increase in procaspase-11 in whole lung (**Figure 4A**) and alveolar macrophages (**Figure 4B**). MTV after Poly(I:C) also resulted in a large increase in the appearance of cleaved caspase-11 in whole lung (**Figure 4A**) and alveolar macrophages (**Figure 4B**). Overall levels of procaspase-1 were not influenced by either stimuli alone (data not shown). There was a slight increase in cleaved caspase-1 after Poly(I:C) but not MTV and this was greatly increased in the combined protocol in both whole lung (**Figure 4A**) and alveolar macrophages (**Figure 4B**). Since caspase-11 may affect caspase-1 activity (30), we repeated these experiments in alveolar macrophages from caspase-11-/- mice and noted that intrapulmonary cleaved caspase-1 levels were significantly lower in both Poly(I:C) and Poly(I:C)+MTV in caspase-11-/- mice compared to WT mice (**Figure 4C**). Nonetheless, cleaved caspase-1 was still induced in caspase-11-/- mice underscoring the partial interdependence of caspases-1 and -11.

We assessed the activation of caspase-11 in advanced glycosylation end product-specific receptor (RAGE) knock-out mice. **Supplementary Data 1** showed that the pro-caspase-11 expression was decreased by block of RAGE after Poly(I:C)+MTV, and activation of caspase-11 was significantly inhibited in RAGE-/- mice, indicating that gut-lung axis pathway may depended on RAGE.

**Figure 5:**
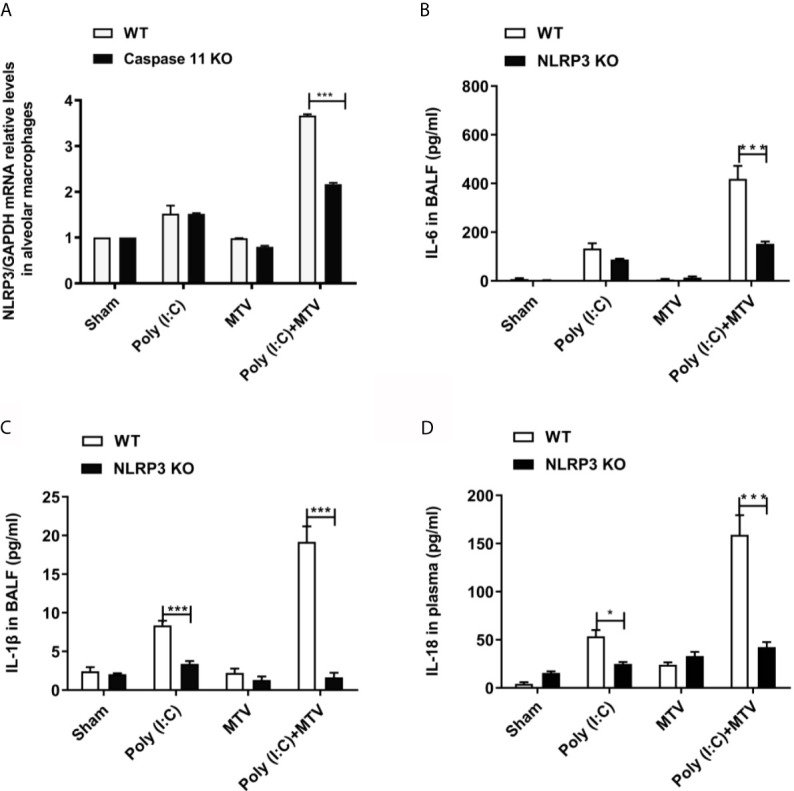
NLRP3 was required for Poly(I:C)-MTV induced lung injury. Poly(I:C)-MTV increased NLRP3 mRNA levels in alveolar macrophages in WT mice but was partially inhibited by caspase-11 KO mice, (n=12) **(A)**. NLRP3 was required for Poly(I:C)-MTV induced IL-6 **(B)**, IL-1β release in BALF **(C)** and IL-18 **(D)** secretion in plasma. In **(B–D)**, n=6 of each group for WT mice, n=8 for NLRP3^-/-^ mice. Results are shown as means ± SEM and compared by one-way ANOVA and Student-Newman-Keuls test. ***p < 0.001.

NLRP3 is part of a common canonical inflammasome that includes apoptosis-associated speck-like protein (ASC), caspase activation and recruitment domain (CARD) and caspase-1 (21). **Figure 5A** shows a significant increase in NLRP3 mRNA level in alveolar macrophages in Poly(I:C) group compared to sham group. There was no difference between the sham and MTV groups. The combination of Poly(I:C) and MTV greatly increased NLRP3 mRNA and this was partially sensitive to genetic ablation of caspase-11 potentially placing caspase-11 upstream of this canonical inflammasome. The role of NLRP3 was further assessed by measuring IL-6 (**Figure 5B**) and IL-1β (**Figure 5C**) secretion in BALF and IL-18 (**Figure 5D**) release in plasma of wildtype and NLRP3-/- mice. As expected, increases in these cytokines after Poly(I:C) and combined Poly(I:C)-MTV were sensitive to ablation of NLRP3. These data underscore the interdependent role of caspase-11 and caspase-1 in combined Poly(I:C)-MTV acute lung injury.

### Caspase-11 Cleavage of GSDMD and Poly(I:C)-MTV ALI Including Pyroptosis in Freshly Isolated Alveolar Macrophages From Injured Lung

**Figure 6:**
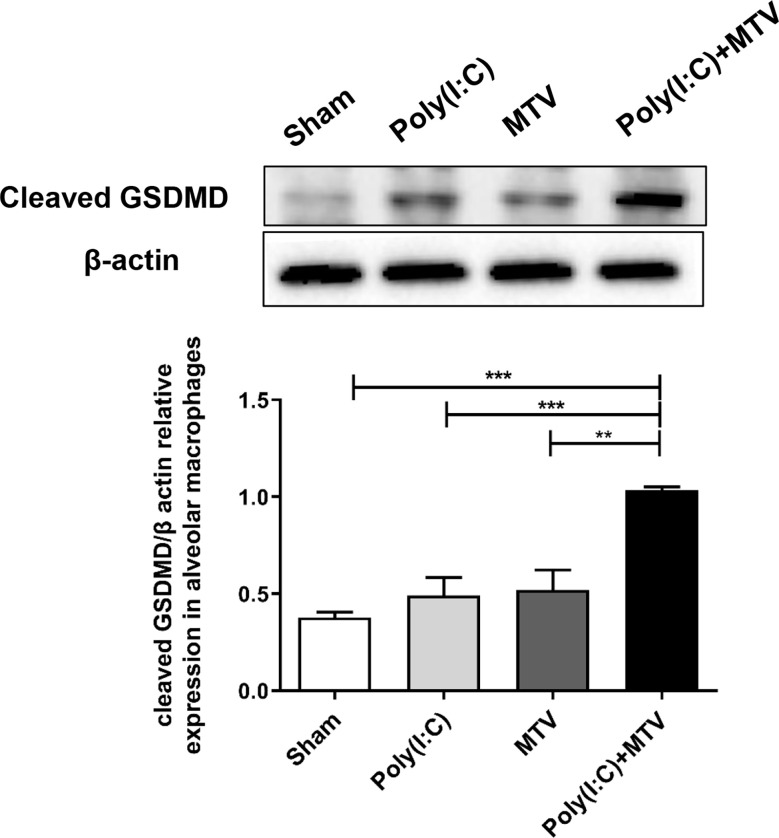
Alterations of protein levels of cleaved GSDMD in alveolar macrophages following Poly(I:C)-MTV. Compared to the sham group, there was no significant increase in GSDMD expression in alveolar macrophages from mice treated with Poly(I:C) or MTV alone. MTV following Poly(I:C) instillation resulted in a significant increase of cleaved GSDMD in alveolar macrophages. Results are shown as means ± SEM (n=4) and compared by one-way ANOVA and Student-Newman-Keuls test. **p < 0.01, ***p < 0.001.

Activation of inflammatory caspases-11 and -1 may lead to cleavage mediated activation of GSDMD that in turn is an obligatory step in inflammatory programmed cell death (pyroptosis). We quantified expression and regulation of GSDMD in alveolar macrophage by Western blot after Poly(I:C) and/or MTV (**Figure 6**). MTV after Poly(I:C) resulted in a significant increase in appearance of cleaved GSDMD in alveolar macrophages (**Figure 6**).

**Figure 7:**
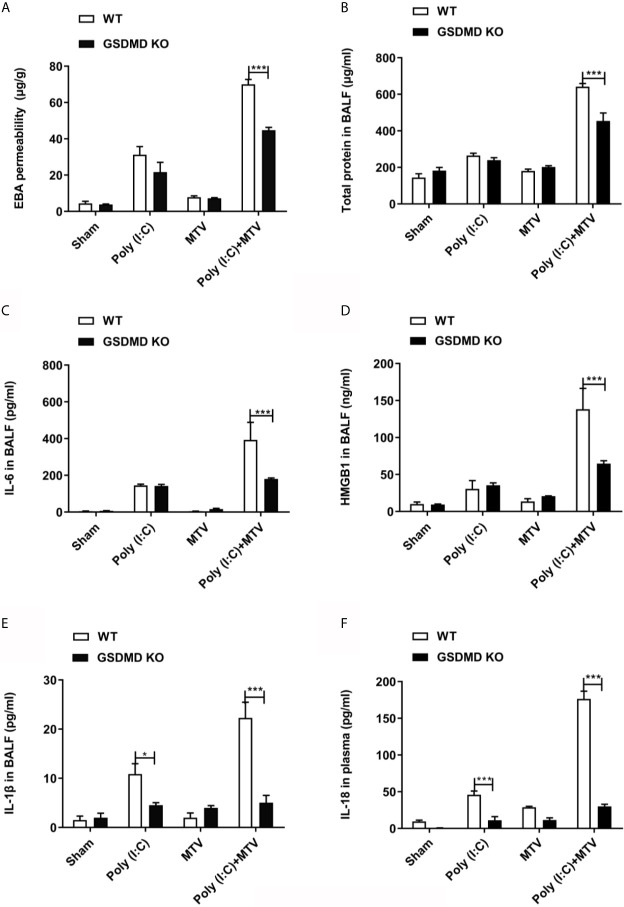
GSDMD was required for Poly(I:C)-MTV induced lung injury. Poly(I:C)-MTV increased EBA permeability, **(A)**, total protein **(B)**, IL-6 **(C)**, HMGB1 **(D)**, and IL-1β **(E)** in BALF as well as IL-18 levels **(F)** in plasma in WT but were inhibited in GSDMD KO mice. n=5 of each group for WT mice and GSDMD^-/-^ mice. Results are shown as means ± SEM and compared by one-way ANOVA and Student-Newman-Keuls test. ***p < 0.001.

Accordingly, we repeated experiments with Poly(I:C), MTV and their combination in GSDMD-/- mice and noted GSDMD sensitive Poly(I:C)-MTV mediated lung injury [as determined by evans blue permeability (**Figure 7A**) and protein in BALF (**Figure 7B**)] and increased cytokine release (**Figures 7C–F**).

**Figure 8:**
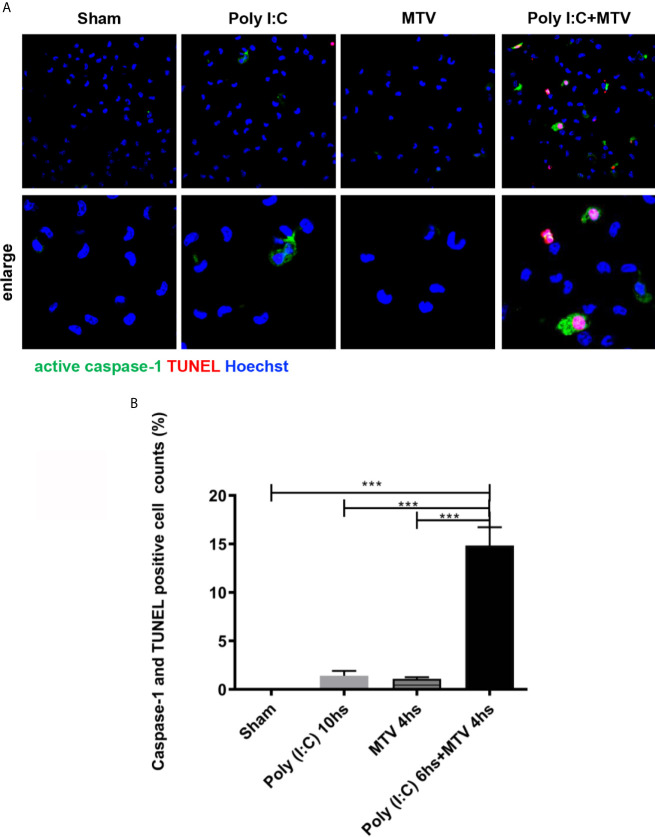
MTV induced alveolar macrophage pyroptosis after Poly(I:C) priming. Alveolar macrophages on and maturation and release of cytokines in combined Poly(I:C)-MTV injury model. The gut-lung axis resulted in activation of pulmonary non-canonical (cytosolic mediated caspase-11 activation) and canonical (Caspase-1) inflammasome (NLRP3) mediated ALI in this two hit model resulting in GSDMD sensitive alveolar capillary barrier disruption, pyroptosis (in alveolar macrophages) and cytokine maturation and release (IL-1β; IL-18) were isolated immediately after mice scarification and adhere for at least 2 hours before staining. Caspase-1 activation was labeled with FLICA caspase-1 and DNA fragmentation was labeled with TUNEL by confocal microscopy **(A)**. Quantification was done by Image J **(B)**. Results are shown as means ± SEM (n=12) and compared by one-way ANOVA and Student-Newman-Keuls test. ***p < 0.001.

GSDMD sensitive injury is consistent with pyroptosis and thus we determined whether pyroptosis occurred in freshly isolated alveolar macrophages from wildtype mice after Poly(I:C), MTV or their combination (**Figure 8**). Macrophages, neutrophils and lymphocytes in BALF were harvested to assess the cell recruitments by Poly(I:C)/MTV. Here we show a significant increase of total cell numbers in BALF in Poly(I:C)+MTV group compared that in sham, Poly(I:C) or MTV group, and the neutrophils contributed the most increase (78%) in the BALF (**Supplementary Data 2**).

Alveolar macrophages were harvested at end of exposure and stained with Alexa Fluor 488-labeled caspase-1 FLICA, Alexa Fluor 546-labeled *in situ* cell death reagent-TMR and Hoechst dye (**Figure 8A**). Quantitation of colocalization of caspase-1 and TUNEL positive cells showed slight increase in pyroptosis after either Poly(I:C) or MTV and a very large increase after combined exposure *in situ* (**Figure 8B**).

## Discussion

**Figure 9:**
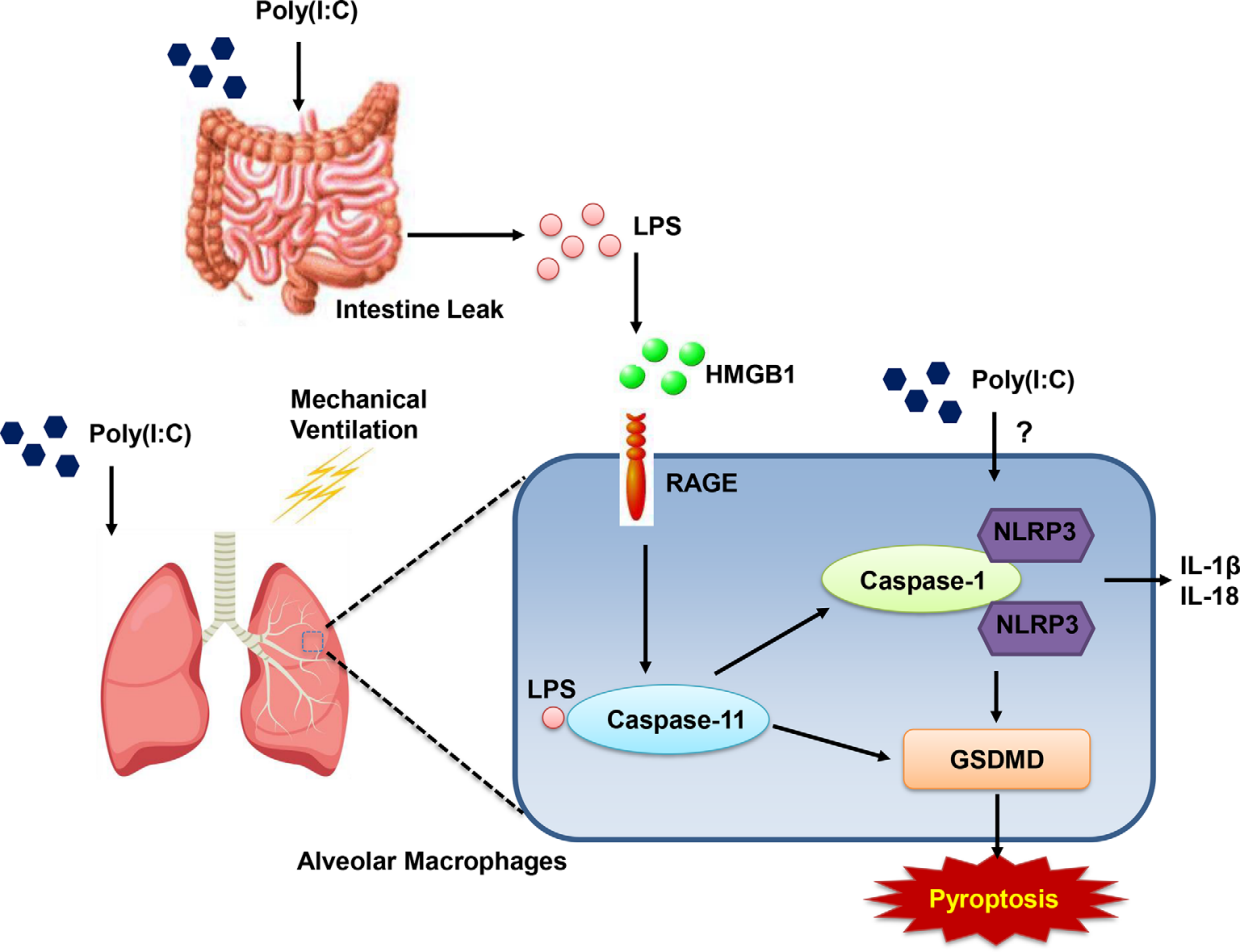
Schematic graph of gut-lung axis. Combined (Poly(I:C)-MTV) insult results in increase in gastrointestinal permeability and endotoxin in plasma and BALF. Poly(I:C)+MTV insult was sensitive to Caspase-11 deletion with no further contribution of caspase-1 but led to large increases in procaspase 11 and its cleaved product as well as cleaved product of caspase-1. Genetic ablation of Gasdermin D (GSDMD) attenuated alveolar-capillary disruption.

We note that otherwise non-injurious moderate tidal volume ventilation exacerbates ALI after i.t. Poly(I:C) in intact mice. Disruption of the alveolar-capillary barrier (**Figures 1** and **2**) in this two-hit model, as previously reported by Chun et al. (21) and us (20), is a central feature of ALI and thus provides a useful preclinical framework in identifying pathways that underscore the major contribution of sepsis to ARDS. Although epidemiologic studies suggest that direct (e.g. pneumonia) sepsis comprises a large component of the risk factors in the development of ARDS, a less understood multifactorial extrapulmonary (indirect or systemic) sepsis is also important (2). In the current study, we note (**Figure 1**) that combined Poly (I:C)-MTV enhanced permeability of gastrointestinal tract and introduced endotoxin to the vascular space and lung. The association of systemic sepsis and ALI (via gut-lung axis) was reinforced by intracellular activation of caspase-11 in the lungs (**Figure 4A**) and alveolar macrophages (**Figure 4B**). Systemic endotoxemia also activated caspase-1 and non-canonical activation of inflammasome (**Figure 5**) with synthesis and release (**Figures 3**, **5** and **6**) of IL-1 cytokines (IL-1β and IL-18) and HMGB1. Interactions between caspase-11 and caspase-1 led to GSDMD dependent barrier disruption (**Figure 7**) and pyroptosis in alveolar macrophages (**Figure 8**). A schema outlining these pathways is presented in **Figure 9** and underscores the complexities of direct lung injury combined with extrapulmonary sepsis.

Mechanical ventilation is a common clinical strategy to rest injured lung and improve gas exchange in critical care setting (3) and to deliver anesthetic agents intraoperatively. The proinflammatory and injurious nature of high tidal volume ventilation has led to adherence to lung-protective ventilation protocols minimizing ventilator induced lung injury (VILI) and greatly reducing morbidity and mortality in ARDS (2–4). Nonetheless, otherwise non-injurious lower tidal volume ventilation may exacerbate preexisting acute lung injury due to bacterial (5, 6) or viral (11) infection, intratracheal endotoxin (7–10) or sterile injury such as hyperoxia (14) or acid instillation (12, 13). Sensitization of VILI to events originating at distal sites or plasma space including exogenous endotoxin (16, 17) or polymicrobial sepsis (18, 19) has also been documented. We confirmed (20) that MTV exacerbated modest acute lung injury secondary to intratracheal instillation of Poly(I:C). Poly(I:C), a TLR3 ligand, is a synthetic analog of double stranded RNA that can be produced by many viruses during their replicative cycles (31). The precise mediators or pathways underlying the synergistic effect of Poly(I:C) and MTV are unknown but are largely independent of TLR4 (21). In the current study, we add the possibility that extrapulmonary endotoxemia secondary to enhanced gastrointestinal permeability with Poly(I:C)-MTV underlies this synergistic effect and reveal a role for intracellular LPS mediated caspase-11 activation, a non-canonical inflammasome pathway and interactions of caspase-11 and caspase-1 in lung injury.

The influence of interactions between gut and lung microbiota in respiratory health is firmly established in chronic (32) and acute (23, 24) disease. Evidence of such microbial mingling in ARDS has suggested therapeutic strategies for ARDS of probiotics (33), novel bio-engineered delivery systems (34) and antimicrobial agents (23). In the current study, combined injury led to an increase in gut permeability with the introduction of endotoxin to the circulation and lung (**Figure 1**). We did not attempt to identify mediators released from lung that might account for this effect on the gut but it is noteworthy that macrophages isolated from Poly(I:C)-MTV treated mice (20) release tumor necrosis factor (TNF)-α and anti-TNF-α antibodies have been shown to abrogate the increase in gut permeability (and lung edema) in high volume ventilation in rats (35). We assumed that endotoxemia in our model was secondary to increased gut permeability as Poly(I:C) mixture was endotoxin free and neither intubation nor circuitry for mechanical ventilation introduced significant amounts of endotoxin in lung (**Figure 1D**). The identification of systemic endotoxemia in the combined Poly(I:C)-MTV protocol motivated us to pursue caspase-11 mediated pyroptosis in lung as Chun et al. (21) reported a TLR-4 independent pathway in this model and Cheng et al. (25) noted that introduction of systemic endotoxin caused TLR-4 independent, caspase-11 mediated pyroptosis in mice. HMGB1 is known to deliver extracellular LPS *via* RAGE to cytosolic caspase-11 (36). We observed elevated levels of HMGB1 after Poly(I:C)-MTV and implicate RAGE in the pulmonary changes after Poly(I:C)-MTV by showing that RAGE-/- mice fail to increase procaspase-11 and cleaved caspase-11 in lungs of mice subjected to Poly(I:C)-MTV (**Supplementary Data 1**). Future experiments to neutralize systemic endotoxemia or eliminate of gut microbes in general (e.g. gnobiotic mice or combined antibiotic therapies) will help advance gut-lung axis hypothesis beyond associative observations in the current study.

Pyroptosis is an inflammatory programmed cell death pathway activated by murine caspase-1 or caspase-11 (caspase-4 and -5 are human orthologs) and requires cleavage and activation of pore-forming effector protein, GSDMD (27). It appears to be a key component of innate immunity and teleologically is an effective means of eliminating intracellular pathogens and signaling host *via* release of inflammatory mediators (25). Nonetheless, excessive activation is implicated in human diseases including sepsis (37). For example, dihydromyricetin, an inhibitor of NLRP3, alleviated cecal ligation and puncture-induced lung histopathologic injury in mice (38). As cell death, per se, and inflammatory mediators are essential components of disruption of alveolar capillary barrier in ALI and ARDS, insight into relevant pathways may be informative of pathogenesis and therapeutic strategies. Caspase-1 activation is well known to be activated *via* a canonical inflammasome pathway (including but not limited to NLRP3) as well as a caspase-11 mediated non-canonical inflammasome pathway (27). Canonical activators include dsRNA [and mechanical ventilation (39)] as well as bacteria; non-canonical activators include gram negative bacteria. Accordingly, there is interaction of these caspases in the maturation and release of cytokines of IL-1 family (IL-1β and IL-18), as well as pyroptosis (27, 30). In the current study, Poly(I:C) is likely to activate the canonical pathway as noted by: a) increase in mRNA of NLRP3 (**Figure 5A**) in alveolar macrophages of Poly(I:C)-MTV treated mice that was only partially sensitive in the caspase-11 null mice; and b) synthesis and release of IL-1β, IL-6 and IL-18 that was sensitive to genetic deletion of NLRP3 (**Figures 5B–D**). Mechanical ventilation has also been shown (39) to activate NLRP3 inflammasome in alveolar macrophages in a caspase-1 dependent fashion underscoring an additional stimulus of canonical pathway in our combined Poly(I:C)-MTV model. Alveolar-capillary barrier disruption was only partially sensitive to genetic ablation of GSDMD (**Figures 7A, B**) as was release of alarmins (HMGB1; **Figure 7D**) whereas release of IL-18 to plasma space (**Figure 7F**) was highly sensitive to GSDMD deletion in combined injury protocol further underscoring the interaction of these pathways and resultant phenotype (**Figure 9**). To the best of our knowledge, *in situ* identification of cellular components of pyroptosis remains challenging in murine tissue. As such, we utilized an ex vivo strategy involving isolation and short-term culture of murine alveolar macrophages from Poly(I:C)-MTV treated mice (**Figure 8**) and quantified pyroptosis *via* co-expression of caspase-1 and TUNEL. Although macrophages (and precursor monocytes) are prototypic of death by pyroptosis, it is noteworthy that other investigators have utilized primary cultures of murine pulmonary endothelial cells isolated from systemic endotoxemic mice (25) or cultured pulmonary epithelial cells (35) directly exposed, *in vitro*, to reveal presence (and differences) in pyroptosis and release of cytokines. Within the limits of our study, we suggest that combined Poly(I:C)-MTV activates both canonical and non-canonical inflammasome pathways involving both caspase-11, caspase-1 and their interaction and GSDMD dependent pyropotosis in at least alveolar macrophages (**Figure 9**). Further cellular origins, aside from alveolar macrophages, awaits improvements in antibody dependent immunohistochemistry in murine lung and pharmacologic separation of caspase-1 and caspase-11 and relevant inflammasome pathways.

In conclusion, By revisiting (25) a two hit model (Poly(I:C)-MTV) of acute lung injury noted to be TLR4 independent, we have detected an additional stimulus, e.g. systemic endotoxemia; as a result of gut-lung axis, both non-canonical caspase-11 [via presumptive cytosolic endotoxemia (28)] and canonical [via NLRP3 inflammasome (38, 39)] and their interactions led to pyroptosis in alveolar macrophages, disruption of alveolar capillary barrier and proinflammatory state within lung. Pharmacologic strategies at disrupting communication between gut and lung, inhibition of inflammasomes or effector molecules (GSDMD) in pyroptosis may be useful in acute lung injury.

## Data Availability Statement

The datasets presented in this study can be found in online repositories. The names of the repository/repositories and accession number(s) can be found in the article/**Supplementary Material**.

## Ethics Statement

The animal study was reviewed and approved by Animal Care and Use Committee.

## Author Contributions

SJ, XD, TB, QL, and L-MZ have made contributions to research concept and design.SJ, XD and MD have made contributions to the acquisition, analysis of data. SJ, HL, BP,TB, and L-MZ have made contributions to data interpretation. CY and WL have made contributions to the creation of new software used in the work. SJ, XL, BP, TB, and L-MZ have made manuscript preparation. SJ, BP, TB, QL, and L-MZ have critically revised of the manuscript and approved manuscript final version. All authors contributed to the article and approved the submitted version.

## Funding

This work was supported in part by the National Natural Science Foundation of China (No. 81772114 to QL, No.81971882 to QL) and Development fund of anesthesiology, Shanghai Pulmonary Hospital (XL) for the design of the study, collection and writing the manuscript, analysis and interpretation of data. Salaries for SJ, XD, CY, WL, XL, and QL were supported by their original hospitals.

## Conflict of Interest

The authors declare that the research was conducted in the absence of any commercial or financial relationships that could be construed as a potential conflict of interest.

## Supplementary Material

Supplementary Data 1 Alterations of protein levels of procaspase-11 and cleaved caspase-11 are dependent on RAGE in the Poly(I:C)-MTV model.MTV following Poly(I:C) instillation resulted in a significant increase in procapase-11 and cleaved caspase-11 in alveolar macrophages from WT mice. The increases were not observed in RAGE KO mice in Poly (I:C)-MTV compared to that in WT mice. Results are shown as means ± SEM (n=4) and compared by one-way ANOVA and Student-Newman-Keuls test. ****p* < 0.001.

Supplementary Data 2 Cell recruitment and classified cell counts in BALF after Poly(I:C)-MTV.MTV following Poly(I:C) instillation resulted in a significant increase of total cell numbers in BALF **(A)**. Neutrophils increased most among all the cells **(B)**. Results are shown as means ± SEM (n=4) and compared by one-way ANOVA. ****p* < 0.001.

## Abbreviations

MTV, moderate tidal volume ventilation; Poly(I:C), polyinosinic-polycytidylic acid; ALI, acute lung injury; BALF, bronchoalveolar lavage fluid; WT, wild type; GSDMD, gasdermin D; NLRP3, nucleotide-binding domain leucine-rich repeat-containing protein 3; RAGE, advanced glycosylation end product-specific receptor; IL, interleukin; HMGB1, high-mobility group box 1; TLR, toll like receptor; ARDS, acute respiratory distress syndrome; VILI, ventilator induced lung injury; LPS, lipopolysaccharide; FITC-D, fluoresceine isothiocyanate dextran; AMs, alveolar macrophages; PBS, phosphate buffered saline; RPMI, roswell park memorial institute; EBA, evans blue albumin; SEM, standard error of mean; H&E, Hematoxylin & Eosin; ASC, apoptosis-associated speck-like protein; CARD, caspase activation and recruitment domain; TNF, tumor necrosis factor.
