# Supplementary figures and images for: Mechanical Ventilation Exacerbates Poly (I:C) Induced Acute Lung Injury: Central Role for Caspase-11 and Gut-Lung Axis
Source: Front Immunol. 2021 Jul 19;12:693874. doi: 10.3389/fimmu.2021.693874 (PMC8327178; doi:10.3389/fimmu.2021.693874)

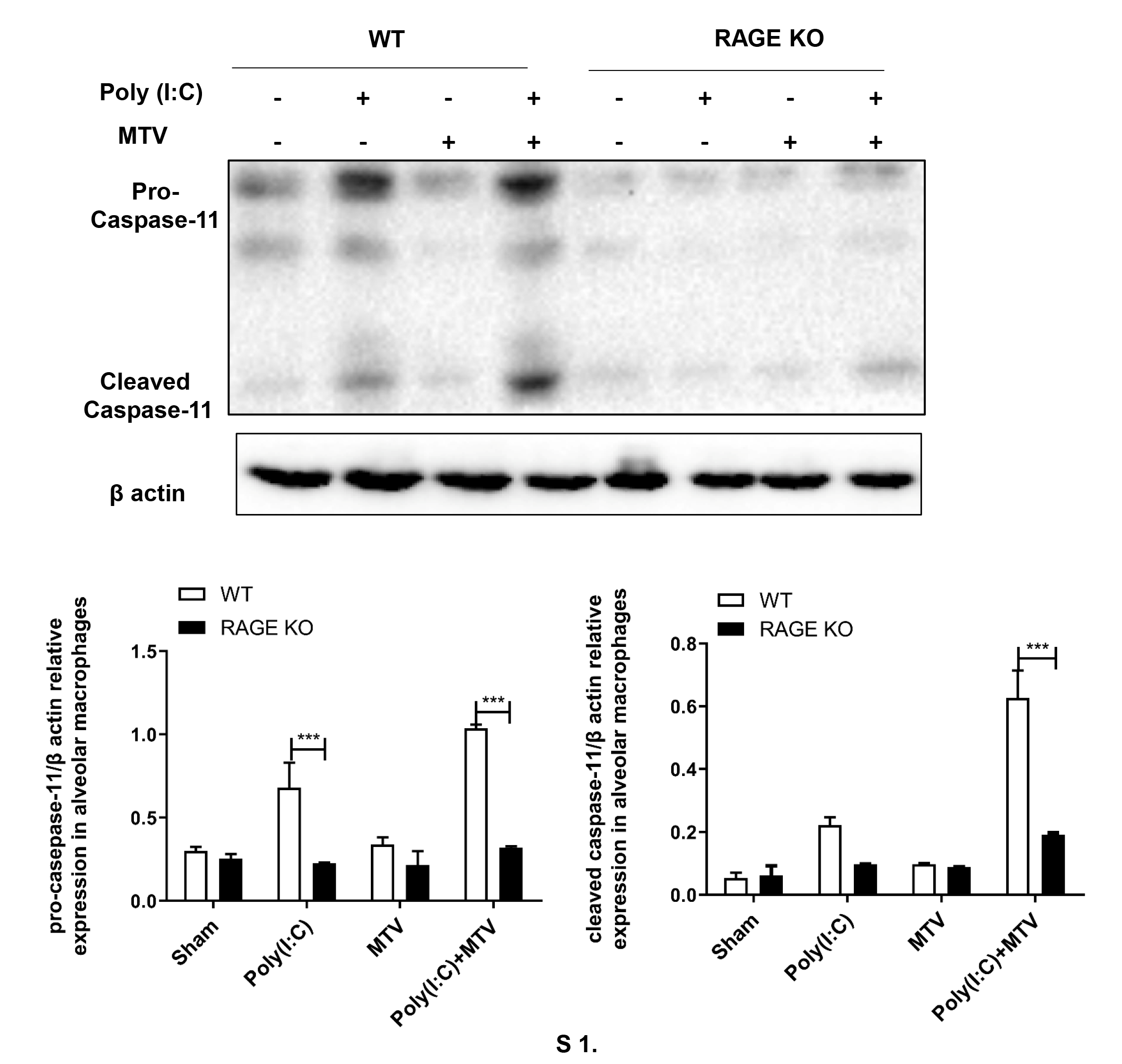

Supplement: Supplementary Data 1 — Alterations of protein levels of procaspase-11 and cleaved caspase-11 are dependent on RAGE in the Poly(I:C)-MTV model.MTV following Poly(I:C) instillation resulted in a significant increase in procapase-11 and cleaved caspase-11 in alveolar macrophages from WT mice. The increases were not observed in RAGE KO mice in Poly (I:C)-MTV compared to that in WT mice. Results are shown as means ± SEM (n=4) and compared by one-way ANOVA and Student-Newman-Keuls test. ***p < 0.001. [file Image_1.tif]

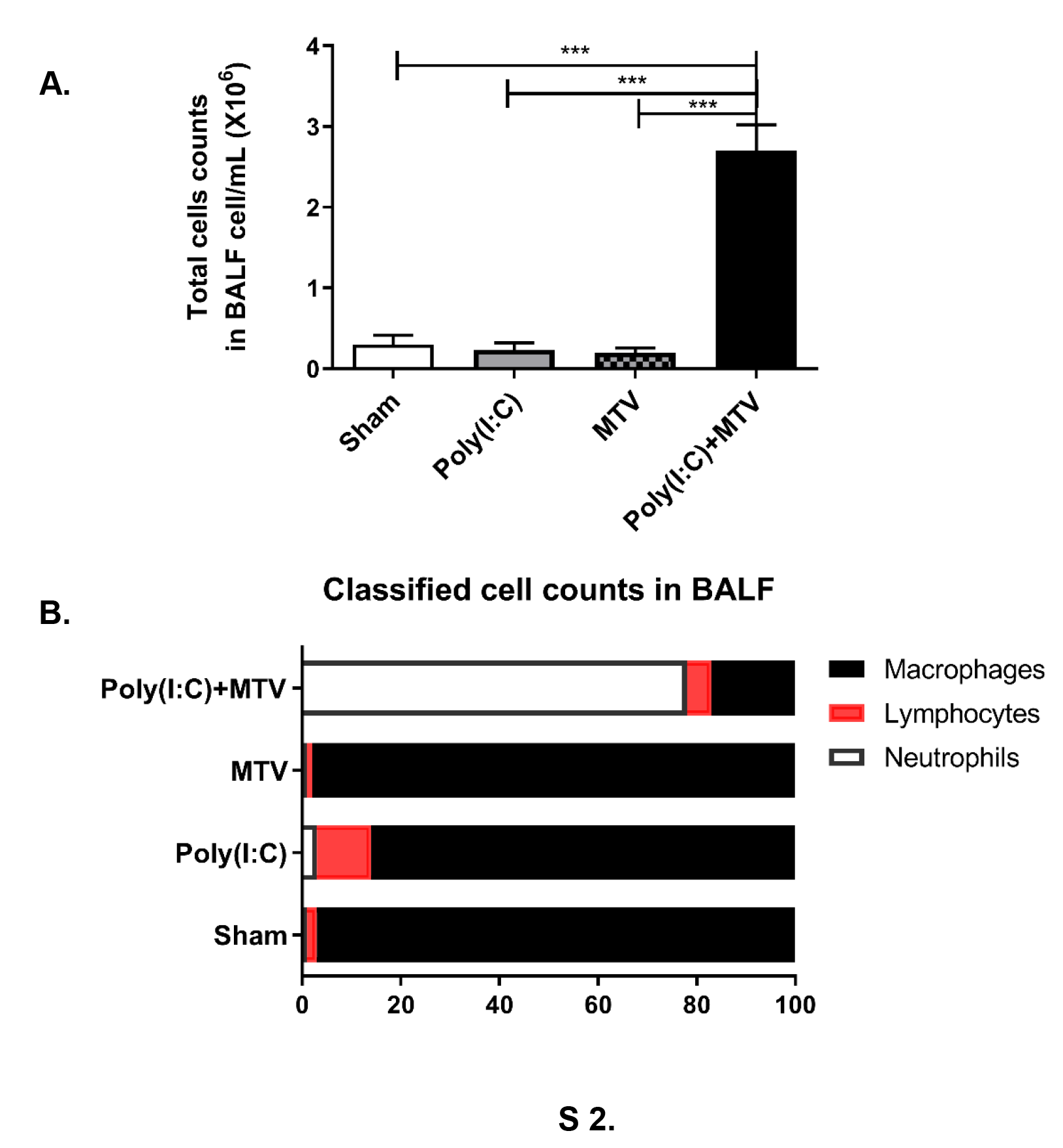

Supplement: Supplementary Data 2 — Cell recruitment and classified cell counts in BALF after Poly(I:C)-MTV.MTV following Poly(I:C) instillation resulted in a significant increase of total cell numbers in BALF (A). Neutrophils increased most among all the cells (B). Results are shown as means ± SEM (n=4) and compared by one-way ANOVA. ***p < 0.001. [file Image_2.tif]
